# The Cell Wall Proteome of *Craterostigma plantagineum* Cell Cultures Habituated to Dichlobenil and Isoxaben

**DOI:** 10.3390/cells10092295

**Published:** 2021-09-02

**Authors:** Gea Guerriero, Charles Achen, Xuan Xu, Sébastien Planchon, Céline C. Leclercq, Kjell Sergeant, Roberto Berni, Jean-Francois Hausman, Jenny Renaut, Sylvain Legay

**Affiliations:** 1 Environmental Research and Innovation Department, Luxembourg Institute of Science and Technology, 5, Rue Bommel, L-4940 Hautcharage, Luxembourg; xuan.xu@list.lu (X.X.); kjell.sergeant@list.lu (K.S.); jean-francois.hausman@list.lu (J.-F.H.); 2 Environmental Research and Innovation Department, Luxembourg Institute of Science and Technology, 41, Rue du Brill, L-4422 Belvaux, Luxembourg; achenc@hotmail.fr (C.A.); sebastien.planchon@list.lu (S.P.); celine.leclercq@list.lu (C.C.L.); 3 TERRA Teaching and Research Center, Gembloux Agro-Bio Tech, University of Liège, 5030 Gembloux, Belgium; Roberto.Berni@uliege.be

**Keywords:** *Craterostigma plantagineum*, dichlobenil, isoxaben, habituation, cell wall, qPCR, proteomics

## Abstract

The remarkable desiccation tolerance of the vegetative tissues in the resurrection species *Craterostigma plantagineum* (Hochst.) is favored by its unique cell wall folding mechanism that allows the ordered and reversible shrinking of the cells without damaging neither the cell wall nor the underlying plasma membrane. The ability to withstand extreme drought is also maintained in abscisic acid pre-treated calli, which can be cultured both on solid and in liquid culture media. Cell wall research has greatly advanced, thanks to the use of inhibitors affecting the biosynthesis of e.g., cellulose, since they allowed the identification of the compensatory mechanisms underlying habituation. Considering the innate cell wall plasticity of *C. plantagineum*, the goal of this investigation was to understand whether habituation to the cellulose biosynthesis inhibitors dichlobenil and isoxaben entailed or not identical mechanisms as known for non-resurrection species and to decipher the cell wall proteome of habituated cells. The results showed that exposure of *C. plantagineum* calli/cells triggered abnormal phenotypes, as reported in non-resurrection species. Additionally, the data demonstrated that it was possible to habituate *Craterostigma* cells to dichlobenil and isoxaben and that gene expression and protein abundance did not follow the same trend. Shotgun and gel-based proteomics revealed a common set of proteins induced upon habituation, but also identified candidates solely induced by habituation to one of the two inhibitors. Finally, it is hypothesized that alterations in auxin levels are responsible for the increased abundance of cell wall-related proteins upon habituation.

## 1. Introduction

*Craterostigma plantagineum* (Hochst.) is a South African angiosperm species belonging to the group of so-called “resurrection plants” which are capable of withstanding extreme dehydration (desiccation) and of recovering fully upon rehydration [1]. The desiccation tolerance of *C. plantagineum*’s vegetative tissues is mediated by the well-known cell wall folding mechanism [2], whereby the cell wall folds in a controlled manner that preserves the integrity of the cell membranes under drought. The desiccation tolerance of *C. plantagineum* is not limited to its leaves, but it is also present in calli, i.e., undifferentiated cells, that can recover from drought after a pre-treatment with abscisic acid (ABA) [3]. Several studies have described the changes in the cell wall composition of resurrection species upon dehydration and rehydration [4,5,6,7,8] and shown the innate structural plasticity towards drought. The key evolutionary strategy to plasticize resurrection species’ cell walls is the occurrence of arabinose-rich polymers [9].

Plant cell walls are dynamic structures and their composition is modified in response to external cues by regulating the expression of cell wall biosynthetic genes [10,11]. Among the cell wall components known to change upon stresses are pectins, which contribute to the cell wall porosity and overall cell-cell adhesion [12,13] and lignins [14]. Plant cell walls’ integrity status is monitored through “sentinels”, such as receptor-like kinases (RLKs), strategically located at the interface between cell wall and plasma membrane and capable of transducing signals to the cells’ interior via their cytoplasmic kinase domain [15,16].

Fundamental aspects underlying the study of plants’ cell wall plasticity have been addressed via the use of cellulose biosynthesis inhibitors (CBIs), which affect the deposition of the main load-bearing component, thereby causing cell wall stress [15,17,18,19]. Both short- and long-term mechanisms were studied and insights into the changes at the transcriptional, proteomic and biochemical levels were obtained [20,21,22,23]. Two of the best characterized and widely used CBIs are isoxaben (IXB) and dichlobenil (DCB) which belong, respectively, to group 1 and 2 of the CBI classification system [24]. CBIs in group 1 cause a clearance of the cellulose synthase complex (CSC) from the membrane focal plane and an accumulation in cytosolic vesicles, while group 2 includes inhibitors that trigger an accumulation of the cellulose synthases (CESAs) at specific foci of the plasma membrane, accompanied by a decrease of the CSC velocity [24].

The various modes of action of CBIs make them an ideal toolbox to understand specificities in the cell wall response of monocots/dicots, or to functionally characterize genes suspected to be involved in cell wall processes, by investigating the response of mutant/overexpressing plants. Plant cells usually show strong phenotypes, such as bulges/swelling [25,26,27], when exposed to CBIs and both monocots and dicots can become habituated to CBIs by compensating the lack of cellulose with other cell wall components [20,22,28,29].

The present study aims at understanding how undifferentiated cells of *C. plantagineum* react when artificial stress conditions are applied to the cell wall. The artificial cell wall stress was triggered by the prolonged exposure to 1 µM DCB and IXB.

The goal is to investigate whether the innate cell wall plasticity of a resurrection dicot species results in similar/different responses to habituation with respect to what is known in the literature. The study is focused on the cell wall response and the techniques used encompass proteomics, as well as optical microscopy and biochemical and gene expression analyses.

## 2. Materials and Methods

### 2.1. Establishment of Calli and Cell Suspension Cultures

Calli were established from *C. plantagineum* leaves propagated in vitro on solid Murashige & Skoog [30] medium (including vitamins, 2.2 g/L; Duchefa Biochemie, Haarlem, The Netherlands) containing sucrose 20 g/L (Sigma-Aldrich, Merck, Darmstadt, Germany) and agar (8 g/L; Kalys, Bernin, France) at pH 5.8. For callus induction, the procedure relying on the use of a buffered glutathione solution described previously by Toldi and colleagues (2002) was followed [31]. Friable calli developing on solid media in darkness were put in liquid medium (same composition as the solid medium, with the absence of agar) to establish cell suspension cultures. The cultures were grown under continuous light at 120 rpm and 26 °C. Subcultures were carried out every ten days (1/10 dilution).

### 2.2. Effects of DCB and IXB and Habituation of Cell Suspension Cultures to the Inhibitors

**Figure 1:**
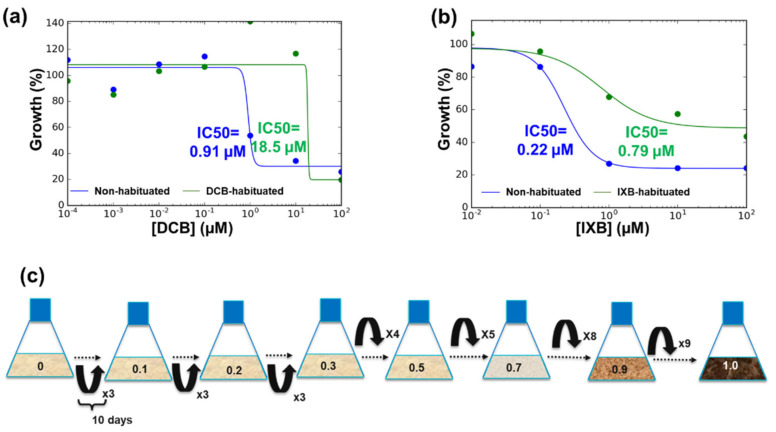
IC_50_ graphs of non-habituated and habituated cells (**a**,**b**) and scheme showing the step-wise adaptation of the cell cultures to DCB and IXB 1 µM (c). The IC_50_ were calculated with the IC_50_ Tool Kit (available at http://www.ic50.tk/index.html accessed date 15 July 2018) and visualized with the Multi-IC_50_ Plotting Tool (available at http://www.ic50.tk/multiic.html accessed date 15 July 2018). Each subculture step in (**c**) lasted 10 days and was repeated for the indicated times (x number) before increasing the concentration (in µM) of the inhibitors (indicated on the flasks).

The effect of DCB and IXB was monitored by assessing the phenotypes with respect to control calli/cells grown on media containing the same concentration of ethanol 0.1% (*v*/*v*) used to dissolve the inhibitors. The cells’ phenotype was analyzed at the optical microscope by observing the aberrant structures (swelling, bulging) in cell suspension cultures exposed to the CBIs. The habituation program consisted in stepwise increases in the inhibitors’ concentrations (0.1–0.2–0.3–0.5–0.7–0.9–1 µM) with an interval of at least 3 cycles of growth before increasing the concentration. Longer intervals (i.e., 5, 8 and 9 cycles of subculture lasting 10 days each) were applied to cultures habituated to higher concentrations (0.5–0.7–0.9–1 µM; Figure 1). The IC_50_ (half-maximal inhibitory concentration, i.e., the concentration of inhibitor required for 50% growth inhibition) in control and habituated cells was calculated by determining the dry weight (DW) of cells (three independent biological replicates were prepared for each concentration). The DW was calculated by pelleting 100 mL of cell cultures at 4000 rpm in a swing-out centrifuge for 10 min at RT and by weighing the cells after drying at 60 °C for 5 days. The % growth was calculated with respect to cells sampled at T0.

### 2.3. Quantification of Cell Wall Monosaccharides and Total Lignin

The extraction and hydrolysis of cell wall materials were performed as described previously [32] on cells habituated to DCB and IXB at 1 µM. Briefly, control, DCB- and IXB-habituated cells (four biological replicates for each condition) were lyophilized and ground to fine powders. About 50 mg of the materials were extracted three times with 80% (*v*/*v*) ethanol for 30 min on ice, followed by a one-time wash with acetone then methanol at room temperature. The residues were treated with 100 U α-amylase (porcine pancreas, Sigma-Aldrich, Merck, Darmstadt, Germany) at 40 °C for 1 h then with an additional 50 U α-amylase for 30 min to remove starch. Cell wall materials were precipitated by four volumes of cold absolute ethanol o/n, followed by three washes with cold ethanol. Five mg of air-dried cell wall materials were hydrolyzed using the two-step Saeman’s hydrolysis procedure (72% sulfuric acid, vortexed intermittently at RT for 1h, then diluted to 10% *w*/*w* and further incubated for 3 h at 100 °C). After hydrolysis, the samples were cooled at room temperature and centrifuged. The supernatant was used for the determination of the monosaccharide composition with high-performance ion chromatography. The monosaccharide composition was determined for each sample in duplicate with a Dionex™ ICS-5000+ Capillary HPIC™ System (Dionex, Thermo Fisher Scientific, Bremen, Germany) by following the same method described in [33].

The lignin content of control, DCB- and IXB-habituated cells (four biological replicates for each condition) was measured using the acetyl bromide spectrophotometric method, as previously described [34,35]. Briefly, 2.6 mL of freshly made acetyl bromide solution (25% *v*/*v* acetyl bromide in glacial acetic acid) was added to 10 mg of cell wall material and incubated for 2 h at 50 °C, followed by an additional hour with vortexing every 15 min. Ten mL of sodium hydroxide (2 M) and 1.75 mL of freshly made hydroxylammonium chloride (0.5 M) were added to the solution. Finally, glacial acetic acid was added to a total volume of 30 mL. The absorbance of the solution was measured at 280 nm with a Lambda 35 UV-Vis spectrophotometer (Perkin Elmer, Waltham, MA, USA).

### 2.4. Gel-Free and Gel-Based Proteomics

The extraction of the cell wall proteins was performed as previously published [36]. The following modifications were made: 2 g PVPP were added to 0.5 g of lyophilized samples and the incubation with 200 mM CaCl_2_ was performed at RT in the presence of 1% (*v*/*v*) protease inhibitor mix (GE Healthcare, Machelen, Belgium). Proteins were quantified with the RC DC™ kit (BioRad, Temse, Belgium). The steps of gel-free proteomics were performed exactly as recently published [37].

Only the proteins identified with a significance Mascot-calculated confidence of 95% were kept for further analysis. The proteins identified with a fold change (FC) > 1.5, a *p*-value < 0.05 and minimum two significant sequences per protein and one unique sequence per protein were considered as differentially abundant proteins.

For 2D-DIGE, the first dimension was run as previously described [36]. Prior to the second dimension, strips were equilibrated 15 min in equilibration buffer (Serva Electrophoresis GmbH, Heidelberg, Germany) complemented with 6 M urea and 1% (*w*/*v*) DTT and further 15 min in equilibration buffer (Serva Electrophoresis GmbH, Heidelberg, Germany) complemented with 6 M urea and 2.5% (*w*/*v*) iodoacetamide (IAA). Strips were then loaded on 2D HPE™ Large Gels NF 12.5% (Serva Electrophoresis GmbH, Heidelberg, Germany) and electrophoresis was carried out using an HPE™ Tower System according to the manufacturer’s instructions. After the front reached the bottom of the gel, the proteins were fixed in a solution containing 15% (*v*/*v*) ethanol complemented with 1% (*w*/*v*) of citric acid at least 2 h and rinsed with MQ water. Gels were subsequently scanned using a Typhoon FLA 9500 scanner (GE-Healthcare, Machelen Belgium) and quantitative analysis was carried out using the Samespots software (v 5.0, Totallab). Spots showing a minimum fold change of 1.5 with an ANOVA *p*-value < 0.01 were selected for picking. MS analyses were performed as previously reported [36]. MS and MS/MS spectra were submitted for database-dependent identification against the in-house transcript database (containing 288,270 sequences [37]), using Mascot. The parameters used were the following: mass tolerance MS 100 ppm, mass tolerance MS/MS 0.5 Da, maximum 2 missed cleavages, fixed modification carbamidomethyl-cysteine, variable modifications oxidation of methionine, oxidation of tryptophan to kynurenine, double oxidation of tryptophan and the loss of 2Da from phenylalanine to didehydrophenylalanine. Proteins were considered identified when at least two peptides passed the Mascot-calculated 0.05 threshold scores. All identifications were manually validated. When high quality MS/MS spectra were not identified in database searches, the sequence of the peptides was determined manually. To obtain an objective confidence score for these peptides, the spectra were resubmitted with adjusted search parameters. When peptides in the same spot matched different database entries, these last were aligned and it was verified whether they belonged to the same protein using the Basic Local Alignment Search Tool-BLAST from NCBI. For the prediction of the subcellular localization (both for the gel-based and gel-free proteomics), the identified proteins were submitted to BLAST against the *Viridiplantae* database. The matching proteins were then submitted to the DeepLoc algorithm with BLOSUM62 encoding [38]. The mass spectrometry proteomics data have been deposited in the ProteomeXchange Consortium via the PRIDE [39] partner repository with the dataset identifiers PXD026723 (gel-based proteomics) and PXD026733 (gel-free proteomics).

### 2.5. RNA Extraction, cDNA Synthesis, and qPCR

RNA extraction was carried out on 5 mL-pelleted control and habituated cells using the RNeasy Plant Mini Kit (Qiagen, Leusden, The Netherlands), coupled to the on-column DNase I treatment. Cells were disrupted using a TissueLyser II (Qiagen, Venlo, The Netherlands) and 5 mm autoclaved stainless steel beads (2 times for 90 s at 50 Hz, using holders previously frozen in liquid nitrogen to avoid thawing). RNA purity/integrity measurements were performed using a NanoDrop ND-1000 spectrophotometer (Thermo scientific, Villebon-sur-Yvette, France) (A260/280 and A260/230 ratios between 1.8 and 2.2) and an Agilent Bioanalyzer (Santa Clara, CA, USA) (RINs were ≥8). One µg of RNA was retro-transcribed using the Superscript II cDNA Synthesis kit (Invitrogen, Carlsbad, CA, USA), following the manufacturer’s instructions. Gene expression analysis was performed in 384-well plates filled with the help of a pipetting robot (EpMotion, Eppendorf, Hambourg, Germany) and using the Takyon Low ROX SYBR Green (Eurogentec, Seraing, Belgium). A melt curve analysis was done at the end of the PCR cycles to verify the primers’ specificity. Primers were designed using the in-house *C. plantagineum* transcriptome. Primer3Plus [40] (available at: http://www.bioinformatics.nl/cgi-bin/primer3plus/primer3plus.cgi accessed date 15 July 2018) was used to design the qPCR primers; the designed primers were checked with the OligoAnalyzer Tool from Integrated DNA Technologies (IDT) (available at: https://eu.idtdna.com/pages/tools/oligoanalyzer accessed date 15 July 2018). The amplification efficiencies were determined with calibration curves prepared using serial dilutions of 6 points (10–2–0.4–0.08–0.016–0.0032 ng/μL). The expression values were calculated with qBasePLUS (version 3.2, Biogazelle, Zwijnaarde, Belgium) by using *TKT3* and *YLS8* as reference genes which were sufficient for data normalization according to geNORM. A total of 4 reference genes were screened: *CAC*, *EIF5A*, *TKT3* and *YLS8* [41]. The details of the primers used are given in Table S1.

### 2.6. Statistical Analyses

The biochemical and gene expression data were log2-transformed and analyzed with IBM SPSS Statistics v20 (IBM SPSS, Chicago, IL, USA). Normality was checked with the Shapiro-Wilk test; homogeneity was verified with the Levene’s test. For normal and homogenous data, a one-way ANOVA was performed followed by the Tukey’s post-hoc test. For data not following normal distribution and/or not homogeneous, a Kruskal-Wallis test was performed together with the Dunn’s post-hoc test.

## 3. Results

### 3.1. Phenotypes of Control and Habituated Cells in Response to DCB and IXB

As a first step, the IC_50_ of control *C. plantagineum* cells was determined for both DCB and IXB and determined to be 0.91 µM and 0.22 µM, respectively (Figure 1a,b, blue lines).

**Figure 2:**
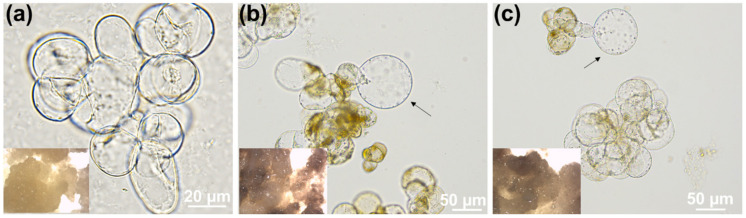
Images of cells and calli (view at the tereomicroscope in the insets) grown under control (**a**), DCB 1 µM (**b**) and IXB 0.2 µM (**c**). The arrows point to abnormally swollen cells.

Cell cultures and calli grown on media containing a concentration of CBIs close to their respective IC_50_ showed abnormal swelling (Figure 2, arrows) and necrotic regions (insets in Figure 2b,c).

A habituation program consisting of a step-wise increase in the concentrations of the CBIs was applied to cell suspension cultures grown in the light (Figure 1c). The cell cultures were kept at a given concentration of the inhibitor for at least three subcultures before further increasing it. At the end of the habituation program, the IC_50_ was measured for the habituated cells and it increased to 18.5 µM and 0.79 µM for DCB and IXB, respectively (Figure 1a,b, green lines).

The effects of DCB and IXB were analyzed both on calli grown on solid media and cell suspension cultures (Figures S1 and S2). *C. plantagineum* calli grown in the presence of DCB and IXB at increasing concentrations showed clear necrosis: the cultures started to develop a brown colour from the concentration of 1 µM DCB and IXB, which then became very intense at the highest concentration of 100 µM (Figures S1 and S2, panels a and c). The calli developed a brown colour too, which became more intense with increasing concentrations of the CBIs (Figures S1 and S2, panels b and d).

**Figure 3:**
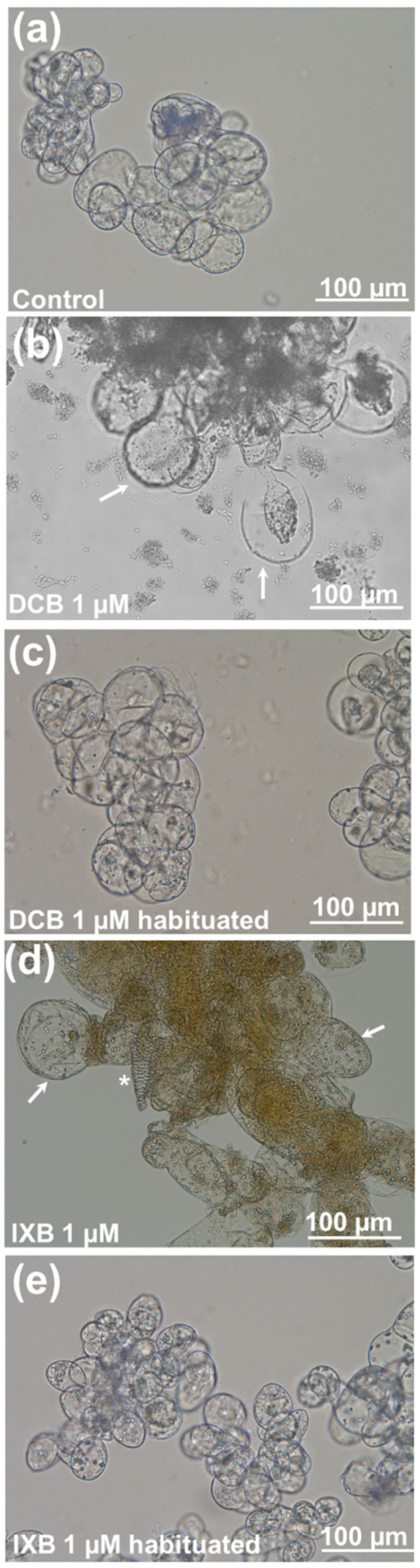
Optical microscope pictures of control *C. plantagineum* cells (**a**), either non-habituated (**b**,**c**) or habituated (**d**,**e**) to DCB and IXB 1µM. The white arrows indicate bulges and swollen regions in the cells. A tracheary element is visible in panel h (*).

Optical microscopy was used to identify any microphenotype in non-habituated cells exposed to the inhibitors and in habituated ones. Control cells had ovoidal shapes with regular contours and were often found in clusters (Figure 3a), while exposure to both DCB (Figure 3b) and IXB (Figure 3c) caused the appearance of bulges and swollen cells (white arrows). Cells habituated to the inhibitors showed instead a regular shape, reminiscent of control cells, without the presence of any bulges nor protrusions (Figure 3d,e).

### 3.2. Biochemical Analyses: Cell Wall Monosaccharide Composition, Total Lignin Content, and Proteomics

**Figure 4:**
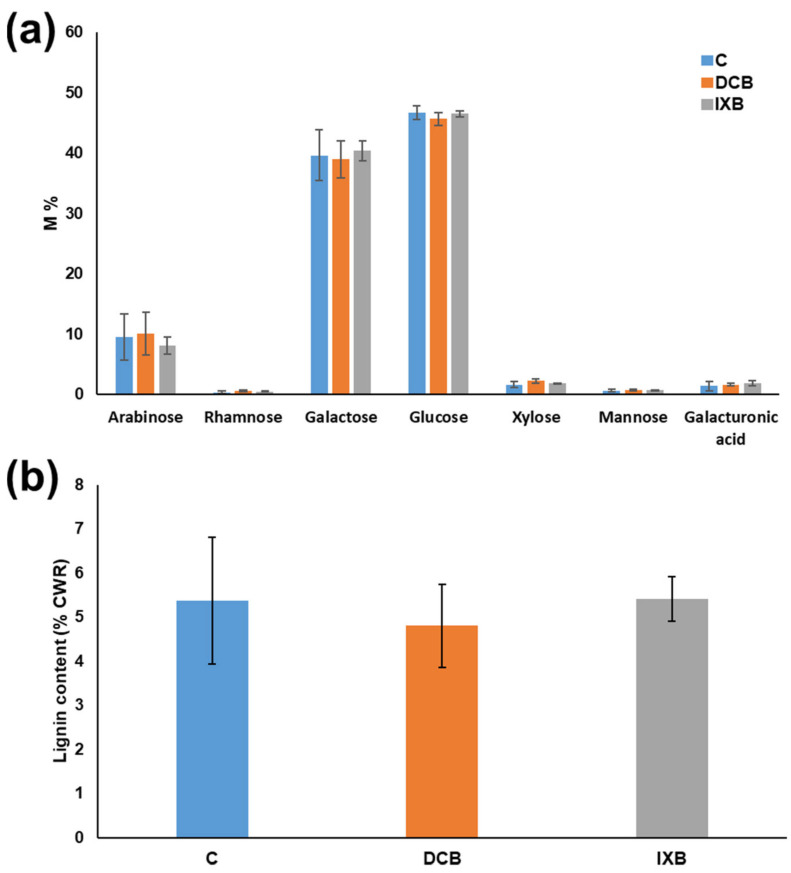
Monosaccharide composition (**a**) and total lignin content (**b**) of the cell wall residue of control (indicated with C) and habituated cells (indicated with DCB and IXB). Values are expressed as the mean ± standard deviation (SD) from four independent biological replicates. No statistically significant differences were computed for all the sugars analyzed (no letters are present on the bars). Arabinose [F(2,9) = 0.30, *p*-value = 0.746], Galactose [F(2,9) = 0.22, *p*-value = 0.810], Glucose [F(2,9) = 1.29, *p*-value = 0.322], Xylose [F(2,9) = 3.02, *p*-value = 0.099], Mannose [F(2,9) = 1.26, *p*-value = 0.328], Rhamnose [*X^2^*(2) = 3.58, *p*-value = 0.167], Galacturonic acid [*X^2^*(2) = 1.42, *p*-value = 0.491], Lignin content [F(2,9) = 0.46, *p*-value = 0.646].

A monosaccharide analysis of the cell wall materials after hydrolysis was performed on control and habituated cells to verify whether habituation resulted in statistically significant changes in the cell wall composition. Besides glucose, expected to be the predominant sugar in the cell wall residue, the chemical analysis revealed a high abundance of galactose, which represented ca. 40% of the total sugar content (Figure 4a). Arabinose contributed to ca. 10% of the total sugars, xylose, and galacturonic acid to ca. 1.5–2%, while rhamnose and mannose accounted for a minority of the total sugar content, i.e., <1%. Habituation to DCB and IXB did not result in statistically significant changes in the abundance of the cell wall monosaccharides (Figure 4a). Likewise, the quantification of the total lignin content revealed no statically significant variations in habituated cells compared to control ones (Figure 4b).

To complement the results on the cell wall monosaccharide composition, proteomics was performed on proteins extracted using a three-step protocol relying on the sequential use of CaCl_2_, EGTA, and LiCl-complemented buffers to sequentially extract proteins with different affinities for cell wall-binding [42]. An approach based on LC-MS and 2D-DIGE-MS was used to obtain information on the changes in abundance of cell wall proteins in response to habituation.

After querying the in-house *C. plantagineum* transcriptome [37], the shotgun approach based on LC-MS identified 310, 254 and 33 significant ID proteins passing the filters [*p*-value < 0.05, maximum fold change (max FC) > 1.5] in the CaCl_2_, EGTA and LiCl fractions, respectively. Of these proteins, 62, 75, and 4 were predicted to be extracellular, corresponding to 20%, 29.5%, and 12% of the total proteins in the 3 fractions (Figure S3 and Supplementary File S1 where the % values appear in the subfolders for each fraction).

Since the objective of this study is the cell wall of *C. plantagineum* cells habituated or not to the CBIs, the focus will be hereafter put on the cell wall proteins (i.e., predicted to be extracellular) identified with proteomics.

**Figure 5:**
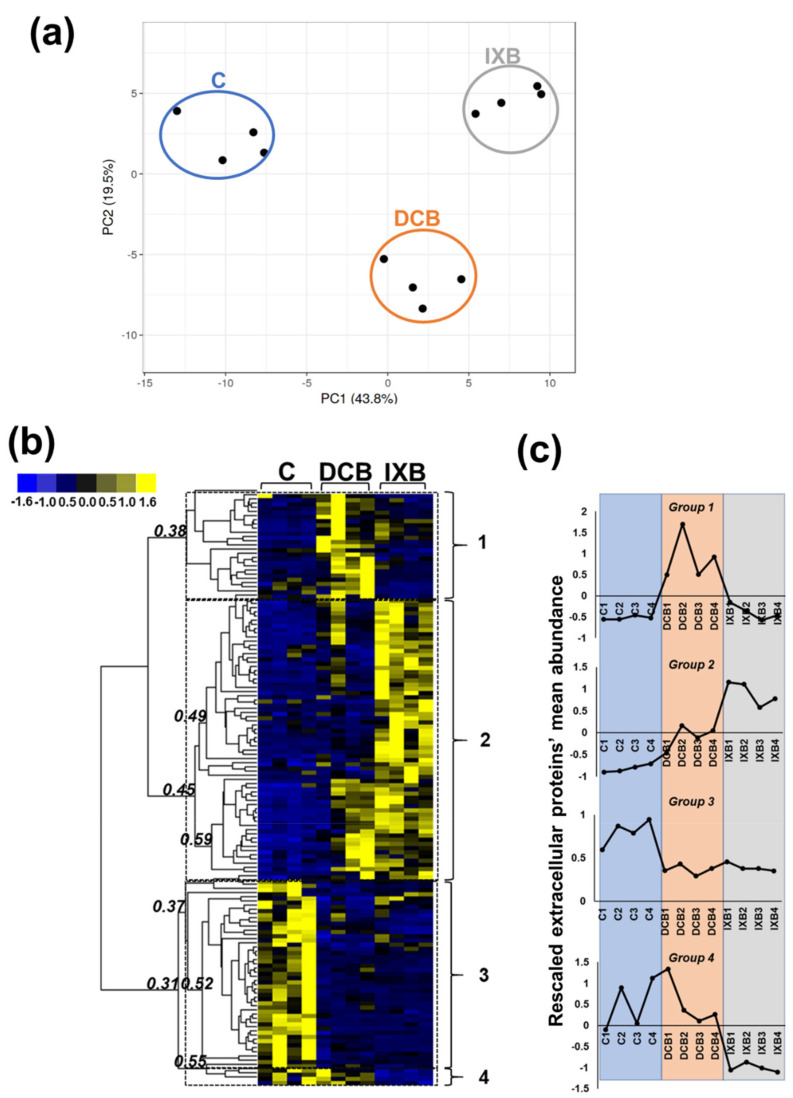
Principal Component Analysis (PCA) in (**a**) of the normalized abundances of extracellular proteins passing the selection filters (*p*-value < 0.05, max FC > 1.5) identified using a gel-free approach in the three fractions. Heatmap hierarchical clustering (HC) in (**b**) of the data used in (**a**) after unit variance scaling [43]. Unit variance scaling was calculated by subtracting from each value the average among all the conditions and dividing by the standard deviation. The numbers in italics in (**b**) indicate the Pearson’s coefficients. The graphs in (**c**) indicate the profiles of the 4 groups determined by setting a correlation coefficient threshold >0.35.

The Principal Component Analysis (PCA) obtained for all the differentially abundant extracellular proteins (141 in total from the 3 fractions) gave a good separation of the three conditions (C, DCB- and IXB-habituated; Figure 5a), thereby indicating a specific cell wall proteomic signature. The normalized abundances visualized as hierarchical clustering (HC) of the heatmaps showed the occurrence of four main clusters with different patterns in the three conditions examined (Figure 5b): the first cluster groups proteins with the highest abundance in DCB-habituated cells, the second those more abundant in IXB-habituated cells, the third is characterized by proteins decreasing in abundance after habituation to the drugs and the fourth includes proteins decreasing in abundance after IXB-habituation. The first group consists of 25 proteins, the second of 67, the third of 45, and the last of only 4 (Supplementary File S1).

A pairwise comparison in terms of FC values was performed on DCB- and IXB-habituated cells vs. control (Table 1 and Table 2) with a threshold set for differences ≥8 (log2 FC ≥ 3). The habituation to the two CBIs resulted in the upregulation of the same set of proteins: among the most abundant ones (log2 FC > 6), it was possible to identify a lignin-forming anionic peroxidase, an alpha expansin 2, a beta-xylosidase 6, and a ubiquitin-like protein.

Common proteins were also present among those showing a 3 ≤ log2 FC < 6, namely a beta-galactosidase 9 and 40-like, an F-box/kelch-repeat protein, an epidermis-specific glycoprotein EP1-like, a desiccation-related protein PCC13–62-like, a cysteine-rich rehydration-responsive 1. Despite these similarities, specific proteins were selectively induced by one of the two inhibitors. An apyrase 2-like protein was induced by DCB (*p*-value = 0.0001 for the comparison control vs. DCB and *p*-value = 0.051 for the comparison control vs. IXB). On the other hand, a leucine-rich repeat (LRR) extensin-like protein 2 (*p*-value = 0.57 for the comparison control vs. DCB and *p*-value = 0.017 for the comparison control vs. IXB), a non-specific lipid-transfer protein 2-like (*p*-value = 0.26 for the comparison control vs. DCB and *p*-value = 0.002 for the comparison control vs. IXB) and a peptide-N4-(*N*-acetyl-beta-glucosaminyl)asparagine amidase A-like (*p*-value = 0.58 for the comparison control vs. DCB and *p*-value = 0.008 for the comparison control vs. IXB) were induced by IXB.

Among the proteins showing high abundance in control cells compared to both DCB- and IXB-habituated ones there were an endochitinase EP3-like and a hevamine A, as well as a polygalacturonase-1 non-catalytic subunit beta-like, a laccase 15-like and a RALF-like 33 protein (Supplementary File S1).

The gel-based approach identified 421 proteins with a *p*-value < 0.01 and max FC ≥ 2; in the different fractions, a total of 220 (CaCl_2_), 171 (EGTA) and 63 (LiCl) spots were identified (Supplementary File S2). Of these, 105, 125, and 48 were predicted to be extracellular, corresponding to 47.7%, 73%, and 77.8% of the total proteins in the three fractions (Figure S4 and Supplementary File S2 where the %values appear in the subfolders for each fraction). As done for the gel-free approach, the emphasis will be hereafter given to the description of the extracellular proteins identified.

The analysis of the differentially abundant extracellular proteins detected with 2D-DIGE confirmed that the habituation to the drugs resulted in the increased abundance of a common set of proteins (Supplementary File S2). Among the top-ranking common proteins (log2FC > 3), there were an epidermis-specific secreted glycoprotein EP1-like and an LRR receptor-like protein kinase PXC2; among the common upregulated proteins with a 2 < log2FC < 3, it was possible to identify a polygalacturonase inhibitor and a germin-like protein 5–1 (Supplementary File S2). A comparison of the proteins showing a log2FC > 2 and identified with the 2 proteomics approaches confirmed that DCB habituation resulted in the increased abundance of an alpha-expansin 2, an EP1-like secreted glycoprotein and a peroxidase 73-like, while IXB habituation induced an EP1-like secreted glycoprotein and a subtilisin-like protease SBT1.9 (Table 1 and Table 2, Supplementary Files S1 and S2).

### 3.3. Gene Expression Analysis of Some Candidates Identified with Proteomics

**Figure 6:**
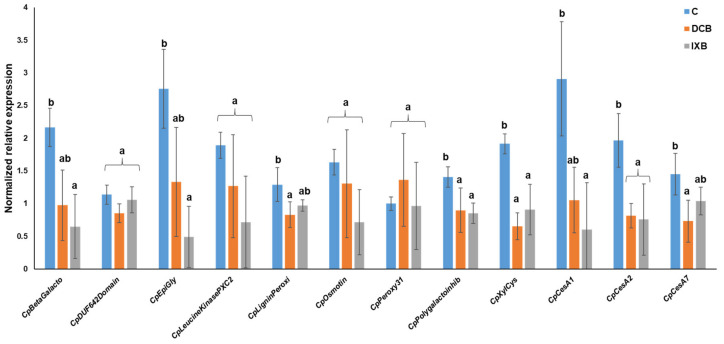
Gene expression analysis of control (indicated with C) and habituated cells (indicated with DCB and IXB). Values are expressed as the mean ± standard deviation (SD) from four independent biological replicates. Different letters indicate statistically significant differences (*p*-value < 0.05) at the one-way ANOVA with Tukey’s post-hoc test or at the Kruskal-Wallis with Dunn’s post-hoc test. *CpBetaGalacto* [F(2,9) = 7.00, *p*-value = 0.015], *CpDUF642Domain* [F(2,9) = 3.57, *p*-value = 0.072], *CpLigninPeroxi* [F(2,9) = 5.21, *p*-value = 0.031], *CpPeroxy31* [F(2,9) = 0.51, *p*-value = 0.618], *CpXylCys* [F(2,9) = 10.04, *p*-value = 0.005], *CpEpiGly* [X^2^(2) = 8.35, *p*-value = 0.015], *CpLeucineKinasePXC2* [X^2^(2) = 3.73, *p*-value = 0.155], *CpOsmotin* [X^2^(2) = 3.50, *p*-value = 0.174], *CpPolygalactoinhib* [X^2^(2) = 6.58, *p*-value = 0.037], *CpCesA1* [F(2,9) = 7.53, *p*-value = 0.012], *CpCesA2* [F(2,9) = 8.35, *p*-value = 0.009], *CpCesA7* [F(2,9) = 6.05, *p*-value = 0.022].

The proteomic analysis was complemented by a targeted gene expression analysis on candidate genes coding for some of the differentially abundant proteins identified with the gel-based and gel-free approaches. Two genes (*CesA*s) coding for cellulose synthases involved in primary and one in secondary cell wall biosynthesis were also included. Differently from the abundances reported previously (Table 1 and Table 2, Supplementary Files S1 and S2), qPCR showed that the expression of selected genes decreased in habituated cells (Figure 6). The majority of the genes targeted were expressed at statistically significant higher levels in control cells; this finding may be linked to post-transcriptional changes between transcripts and corresponding proteins. The only exceptions were the genes coding for the DUF642 domain-containing protein, the LRR receptor-like protein kinase PXC2, the osmotin, and the peroxidase 31. Despite not being statistically significant, the trend of the peroxidase 31-encoding gene showed slightly higher values in DCB-habituated cells, similarly to what observed with the protein’s abundance (Supplementary Files S1 and S2).

## 4. Discussion

Habituation to CBIs has been widely used in the literature to identify the changes triggered in response to a decreased production of cellulose in both monocots and dicots [20,21,44,45]. This approach is simple but effective to understand the compensatory mechanisms put in place by cells during division and expansion, in conditions where the production of the cell wall load-bearing component decreases. Molecular studies on habituation to CBIs have been carried out on plant calli and cell suspension cultures, because of the clear advantage of this biological material compared to whole plants: they are sub-cultured periodically on solid/in fresh liquid media; therefore, this procedure provides the required time-frame for habituation to take place. Additionally, morphological changes in texture/color or occurrence of microphenotypes can be easily distinguished in calli [22,28] and, in the case of cell suspension cultures, the exposure to the drug is constant as the cells are immersed in the culture medium.

The literature data documented both morphological and biochemical changes in calli habituated to DCB. Hollow protuberances with larger cells characterized by lamellate cell walls were observed in DCB-habituated bean cells and these aberrant microphenotypes were accompanied by a replacement of the xyloglucan-cellulose network with a pectin-rich one containing polyuronides (mainly homogalacturonans) [22].

The present study was conceived based on *Craterostigma*’s physico-chemical cell wall plasticity, which contributes to its remarkable desiccation tolerance. We used the approach relying on the use of CBIs to get information on *Craterostigma*’s cell wall response to habituation with the goal of comparing it with the already described mechanisms in non-resurrection monocot and dicot species.

*C. plantagineum* calli and cell suspension cultures exposed to 1 µM DCB or IXB showed necrosis and swollen cells (Figure 2 and Figure 3; Figures S1 and S2) and, after the habituation program, the IC_50_ increased almost 20-times for DCB and >3-times for IXB (Figure 1). The occurrence of swollen cells and bulges was already previously reported in thale cress seedlings exposed to 20 nM DCB and IXB [25]. Habituation resulted in the absence of aberrant cell phenotypes when the *Craterostigma* cultures were grown in the presence of DCB and IXB at 1 µM: the cells’ morphology was indeed regular and not vacuolated, a result proving the acquired habituation after prolonged exposure to step-wise increases of the inhibitors (Figure 3).

Despite the clear morphological differences between habituated and non-habituated cells, the biochemical analysis of cell wall monosaccharides did not reveal statistically significant changes in the levels of glucose, nor of the other major monosaccharides detected, i.e., arabinose and galactose (Figure 4a). This result can be explained by the use of concentrated sulfuric acid (72% *w*/*w*) subsequently diluted to 10% (*w*/*w*) to perform the so-called “two-step hydrolysis” which releases glucose from all possible sources, including the tightly-packed crystalline cellulose [46]. Hence, changes in cellulose content can be masked by glucose released from matrix polysaccharides. It was previously reported that maize cells habituated to DCB were characterized by an easier extractability of xyloglucan [21]: we cannot rule out that the habituation of *Craterostigma* to DCB and IXB may also trigger a similar phenomenon, since we did not perform sequential extraction of cell wall components, nor did we use a parallel one-step matrix hydrolysis using directly a diluted sulfuric acid solution (4% *w*/*w*) to infer the quantity of glucose deriving from crystalline cellulose. Concerning the monosaccharides relative to pectins (rhamnose, arabinose, galacturonic acid), no changes were detected in habituated cells, a finding indicating a different response with respect to DCB-habituated bean cell cultures or IXB-habituated *Arabidopsis* cells, in which an increase of pectins was observed [17,23]. Therefore, the common mechanism reported so far and implying changes in pectins’ abundance does not occur in *Craterostigma* habituated cells. It remains to be verified whether the extractability of xyloglucan increases as a consequence of habituation.

Proteomics was previously used to get a broad understanding of the biological processes underlying habituation of maize cells to DCB and to complement gene expression and biochemical analyses [20]. Here, a similar approach was adopted which was selectively focused on the cell wall proteomes of C, DCB and IXB-habituated cells. Both a gel-free and gel-based approach was used to identify the cell wall proteome signatures of habituated cells. While both techniques are reliable, the scope of each is different: gel-free proteomics is more sensitive and allows the detection of lower abundant proteins than a gel-based approach, hence resulting in a lower percentage of cell wall-localized proteins identified in this study. While a gel-based approach is less sensitive, the use of 2D-gels is still the most efficient way to separate intact proteins and thus allows the visualization of covalent modifications of the primary structure of proteins. Among the hits with the highest FC difference, proteomics revealed a common set of cell wall-related proteins involved in stress response that were induced under habituation to both inhibitors (Table 1 and Table 2, Supplementary File S1). A lignin-forming anionic peroxidase showed the highest FC in habituated cells, followed by an alpha-expansin 2 and a beta-xylosidase. The anionic peroxidase identified has between 68–77% identity with tobacco orthologs, which are by far the best characterized in terms of role: they are involved in the polymerization of cinnamyl alcohols in vitro, in cross-linking of feruloylated polysaccharides with extensins [47]. Anionic peroxidases were shown to be induced by auxin and to be involved in stiffening of the cell wall [48]. In *Arabidopsis*, cells treated with inhibitors such as IXB and thaxtomin A were resistant to the auxin efflux transport inhibitor 1-napthylphthalamic acid (NPA) [49] and in zucchini, the anionic lignin-forming peroxidase APRX was shown to respond to auxin levels [50]. Additionally, tobacco plants in which an anionic peroxidase was silenced showed phenotypes reminiscent of plants with altered auxin/cytokinin levels [51] and in zucchini, the anionic peroxidase APRX was shown to have auxin oxidase activity [52]. Therefore, a link between habituation to CBIs and auxin homeostasis may exist and could explain the increased abundance of the anionic peroxidase in habituated *Craterostigma* cells. Cell wall mutants, such as *Radially Swollen 1*, display perturbations in auxin transport, as demonstrated by the altered expression of auxin-responsive and *CesA* genes [53].

The analysis of lignin in *Craterostigma* cells revealed no statistically significant differences between control and habituated cells (Figure 4b). It should however be kept in mind that the anionic peroxidase’s role in lignification was shown to be limited (e.g., in tobacco mutants, in which the total amount of lignin was unchanged). Indeed, anionic peroxidases mediate the deposition of lignin-like polymers in response to an exogenous stress, such as wounding [54], as confirmed by experiments in which their overexpression leads to a higher resistance to biotic stress [55]. The involvement of stress signals in the induction of cell wall-related proteins, such as the anionic peroxidase, is in agreement with the known mode of action of the inhibitors on cell wall integrity [56].

Future analyses on the phenolic compounds produced by control and habituated cells should be performed to understand whether habituation to CBIs is accompanied by changes in the abundance of phenolic acids. Phenolic acids play a role in the structural integrity of plant cell walls, indeed some cross-link cell wall components, thereby affecting expansion, degradation and defense against pathogens [57]. *Craterostigma* is a dicot and feruloylated arabinoxylans are likely not present since they are typically found in type II cell walls; however, the abundance of some phenolic acids, such as ferulic acid, may be affected by habituation and therefore increase the cross-linking of pectic arabinans and galactans, as well as pectins with extensins, thus ultimately affecting the mechanical properties of the cell wall.

The observed higher abundance of the alpha expansin 2 and beta xylosidase 6 can also be linked to changes in auxin levels due to habituation. These proteins are involved in cell wall loosening by means of the acid-growth mechanism [58,59], whereby a plasma membrane H^+^-ATPase, via the phosphatase-inhibiting action of SAURs (SMALL AUXIN UP RNA), acidifies the apoplast and induces cell wall loosening processes, such as those mediated by expansins [60]. As discussed above for the anionic peroxidase, the higher abundance of such cell wall-related proteins may not be strictly indicative of cell expansion, but rather linked to auxin-driven processes. Auxin is known to trigger the induction of transcripts associated with cellulose-, hemicellulose-, and pectin-linked processes [61]. It will therefore be interesting in the future to verify whether changes in this phytohormone occur during habituation in *C. plantagineum*.

The above-described cell wall proteins belong to group 2 according to the HC of the heatmaps (Figure 5). This group consists of proteins highly induced after IXB habituation (and to a lesser extent after DCB habituation). The majority of hits found in this group is cell wall-related (namely peroxidases, alpha/beta-xylosidases, alpha/beta-galactosidases, expansins, extensin-like proteins, endo beta-1,3-glucosidases, a pectin acetylesterase), but stress-related/defense proteins can also be identified. Among these, a low-temperature-induced cysteine proteinase-like, a cysteine-rich rehydration-responsive 1, a desiccation-related protein PCC13-62-like and a germin-like protein subfamily 1 member 13 were identified (Supplementary File S1).

Cysteine proteases accumulated during desiccation in *C. plantagineum* [62] and their role was put in relation to oxidative stress and programmed cell death (PCD) [63]. IXB triggers PCD and this phenomenon depends on *de novo* gene transcription: among the up-regulated transcripts, genes coding for several of the above-mentioned cell wall- and stress-related proteins were identified in *Arabidopsis* cells exposed to IXB [18].

The gene coding for the apoplast-localized cysteine-rich rehydration-responsive 1 protein was reported as a taxonomically restricted transcript, playing a role in normal conditions and in the recovery from dehydration in *C. plantagineum* leaves [64]. A link with osmotic stress management was proposed, since the transcript was induced in osmotically stressed leaves [64]. CBIs damage the cell wall integrity and are known to activate mechanosensitive genes mimicking osmosignaling by hypo-osmotic shock [65]; hence, the higher abundance of the protein can be linked to osmotic stress-related adjustments underlying habituation.

The desiccation related protein PCC13-62-like is stress-related [66] and a *pcC13-62* transcript was induced upon desiccation in *C. plantagineum* [3]. A dehydration-responsive element was identified in the promoter of *pcC13-62* and heterologous expression of *pcC13-62* promoter: GUS in thale cress showed response to salt stress too [67]. The higher abundance of the PCC13-62-like protein in habituated cells is likely related to the need of withstanding a stress status due to the presence of the CBIs.

The germin-like protein identified by shotgun proteomics is not the one reported previously as possessing SOD activity and contributing to cell wall integrity in *C. plantagineum* (CpGLP1) [68]; however, given its increased abundance under habituation, it is legitimate to infer a potential role with cell wall processes under growth with CBIs. The contig corresponding to the protein is partial and an alignment of the protein sequence revealed that the RGD motif likely involved in signal transduction in the extracellular matrix in CpGLP1 is substituted by AGD. It should however be noted that AGD and RGD motifs were shown to be competitive ligands for the integrin receptor [69] and therefore the identified germin-like protein may also mediate signal transduction in the extracellular matrix under habituation.

One result that emerged from this study is the overall lack of correlation between gene expression (Figure 6) and proteomics. The candidates selected for qPCR showed a tendency towards downregulation after habituation and this agrees with the previously reported conclusion that habituation results in the switching off of cell wall- and stress-related mechanisms at the transcriptional level in thale cress cell cultures [18]. Three genes coding for 2 CESAs involved in primary (*CesA1* and *CesA2*) and 1 involved in secondary cell wall formation (*CesA7*) were selected and they showed downregulation in habituated cells. More specifically, *CesA2* was significantly downregulated in both DCB- and IXB-habituated cells (Figure 6), although the abundance of the corresponding protein increased, as detected by shotgun proteomics (EGTA fraction, Supplementary File S1). This result differs from that reported in habituated maize cell cultures, where an increased expression of some *CesA*s was observed [20,21]; maize and *Craterostigma* belong to the monocot and dicot clades, which are characterized by a different composition of the cell wall. Therefore, it is not totally surprising to observe a different behavior in terms of cell wall response. Previously, the abundance of the cellulose synthase celA1 was shown to increase under DCB in tobacco BY-2 cells through a possible stabilizing mechanism preventing proteolytic degradation [70]; long-term exposure to IXB caused the accumulation of CESAs in SmaCCs (small CESA compartments [71]). The increase in CESA detected with gel-free proteomics may be the result of an increased stabilization or increased abundance of SmaCC populations in the habituated cells (Supplementary File S1).

Among the proteins uniquely induced after DCB habituation there was an apyrase 2-like (Supplementary File S1). In thale cress, apyrase genes are involved in the growth, development, and biotic stress response by modulating the levels of extracellular ATP (eATP) [72,73]. Pollen tubes overexpressing an apyrase grew faster by reducing the concentration of extracellular nucleotides; in DCB-habituated cells, the induction of the apyrase 2-like protein can control the release of eATP and may be required for auxin homeostasis, since its accumulation can lead to an increase of growth-inhibitory levels of auxin [72]. As discussed above, habituation may exert an action on cell wall-related proteins via alterations in auxin homeostasis and the higher abundance of the apyrase likely mediates a control over the endogenous levels of auxin.

The shotgun proteomics analysis allowed to identify a peptide-N4-(*N*-acetyl-beta-glucosaminyl)asparagine amidase A-like (PNGase-like), an LRR extensin-like protein 2 and a non-specific lipid-transfer protein 2-like as most abundant in IXB-habituated cells. The role of the cell wall PNGase can be in relation to the production of free *N*-glycans, which are known to be signaling molecules involved in growth and development [74,75], while the LRR extensin-like protein 2 and non-specific lipid transfer protein are related to the sensing of the cell wall integrity and cell wall organization [76,77].

The gel-based approach allowed the identification of 454 spots showing statistically significant differences (Supplementary Figure S2). Of those, 278 are predicted to represent extracellular proteins; in particular, DCB- and IXB-habituation induced the accumulation of two proteins with a log2FC > 3 (Supplementary File S2): an epidermis-specific secreted glycoprotein EP1-like (identified in 38 different spots in DCB-habituated cells and 45 in IXB-habituated *Craterostigma*) and the LRR receptor-like protein kinase PXC2 (identified in 9 spots in DCB-habituated cells). The same higher accumulation was found for both proteins in the gel-free analysis (Table 1 and Table 2 and Supplementary File S1).

The EP1 glycoprotein was first isolated from carrot cell suspension cultures and proposed to be involved in restricting water flow through the outer epidermal cell wall via direct modifications of the wall structure [78]. Its increased abundance in habituated cells may be put in relation with cell walls’ modification following habituation. One clear phenotype in control *C. plantagineum* cells exposed to DCB and IXB is swelling, a reported feature in CBIs-treated plant cells due to the difficulties of a weakened cell wall to regulate turgor pressure. The EP1-like protein can induce modifications of the cell wall in habituated cells (e.g., deposition of water-proof lignin-like polymers) to restrict water influx and therefore control the internal turgor pressure. Habituated cells showed a normal phenotype (no bulges, no swelling; Figure 3) and the EP1-like protein may play an important role in this.

The PXC2 protein was shown to be involved in stress-related aspects (osmotic stress [79]) in thale cress and to play a role in secondary cell wall formation in fibers [80]: its increased abundance in DCB- and IXB-habituated cells can be linked to cell wall modifications increasing osmotolerance in a condition whereby the cell wall is weakened and its integrity altered.

## 5. Conclusions

This study showed that cell suspension cultures of the resurrection species *C. plantagineum* can be successfully habituated to grow in the presence of DCB and IXB; habituation resulted in increased IC_50_ values and coincided with the restoration of normal cell phenotypes. Proteomics using shotgun and gel-based approaches allowed the identification of a common set of proteins induced by both CBIs, but also discriminated some proteins solely induced by one of the two drugs, namely an apyrase-like protein in DCB-habituated cells and a PNGase, an LRR extensin-like protein 2, a non-specific lipid-transfer protein 2-like in IXB-habituated *C. plantagineum*. Habituation of *C. plantagineum* cells showed both similar and different aspects compared to other species. The resurrection species responded to CBIs in a manner that was similar to other reported dicots, e.g., by slowing the growth rate and by showing phenotypes, namely bulges, swelling. The expression of few genes revealed downregulation in habituated cells, thereby confirming the previously reported switching off of cell wall- and stress-related mechanisms. Differently from what is proven for other dicots, sugars related to pectins did not change following habituation; it remains to be verified whether the extractability of xyloglucans is affected following habituation. Finally, taken together, the results obtained with proteomics suggest that habituation in the resurrection species involves auxin-related processes that influence the cell wall.

## Figures and Tables

**Table 1:** Excerpt of Supplementary File S1 showing proteins with higher abundance in DCB-habituated cells compared to the control (C). The fold change (FC ≥ 8) and log2 FC ≥ 3, as well as the contig numbers, group classification according to the HC are provided.

Description	Contig Number	DCB/C FC	DCB/C log2 FC	Group
lignin-forming anionic peroxidase	Cp_V2_contig_18867	7656.2	12.9	2
beta-glucosidase 40	Cp_V2_contig_7909	896.0	9.8	1
AF230277_1alpha-expansin 2	Cp_V2_contig_38601	496.0	9.0	2
probable beta-D-xylosidase 6	Cp_V2_contig_42585	198.6	7.6	2
ubiquitin-like protein	Cp_V2_contig_19299	74	6.2	2
beta-galactosidase 3-like	Cp_V2_contig_34887	56.5	5.8	2
beta-galactosidase 9	Cp_V2_contig_9145	47.4	5.6	1
F-box/kelch-repeat protein	Cp_V2_contig_38461	12.3	3.6	2
apyrase 2-like	Cp_V2_contig_8189	12.1	3.6	1
heparanase-like protein 2	Cp_V2_contig_6154	11.4	3.5	2
epidermis-specific secreted glycoprotein EP1-like	Cp_V2_contig_23179	11.2	3.4	2
peroxidase 73-like	Cp_V2_contig_19463	10.9	3.4	1
putative lipid-transfer protein DIR1	Cp_V2_contig_45594	10.4	3.4	1
beta-glucosidase 40-like	Cp_V2_contig_28315	10.4	3.4	1
desiccation-related protein PCC13-62-like	Cp_V2_contig_37935	9.3	3.2	2
cysteine-rich rehydration-responsive 1	Cp_V2_contig_1534	8.2	3.0	1

**Table 2:** Excerpt of Supplementary File S1 showing proteins with higher abundance in IXB-habituated cells compared to the C. The fold change (FC ≥ 8) and log2 FC ≥ 3, as well as the contig numbers, the group classification according to the HC are provided.

Description	Contig Number	IXB/C FC	IXB/C log2 FC	Group
lignin-forming anionic peroxidase	Cp_V2_contig_18867	7103.8	12.8	2
AF230277_1alpha-expansin 2	Cp_V2_contig_38600	908.6	9.8	2
probable beta-D-xylosidase 6	Cp_V2_contig_42585	333.8	8.4	2
beta-galactosidase 3-like	Cp_V2_contig_34887	99.8	6.6	2
ubiquitin-like protein	Cp_V2_contig_19299	73.3	6.2	2
phylloplanin-like	Cp_V2_contig_30862	42.3	5.4	2
beta-galactosidase 9	Cp_V2_contig_9145	32.6	5.0	1
beta-glucosidase 40	Cp_V2_contig_7909	31.0	5.0	1
cysteine-rich rehydration-responsive 1	Cp_V2_contig_1534	24.2	4.6	2
serine carboxypeptidase-like 42	Cp_V2_contig_44837	24.2	4.6	2
desiccation-related protein PCC13-62-like	Cp_V2_contig_37935	23.5	4.6	2
non-specific lipid-transfer protein 2-like	Cp_V2_contig_21060	22.6	4.5	2
epidermis-specific secreted glycoprotein EP1-like	Cp_V2_contig_2034	21.7	4.4	2
Elongation factor 1-alpha	Cp_V2_contig_23179	17.7	4.1	2
leucine-rich repeat extensin-like protein 2	Cp_V2_contig_13652	16.8	4.1	2
probable beta-D-xylosidase 7	Cp_V2_contig_29850	15.2	3.9	2
low-temperature-induced cysteine proteinase-like	Cp_V2_contig_1674	14.0	3.8	2
heparanase-like protein 2	Cp_V2_contig_6154	13.1	3.7	2
probable purple acid phosphatase 20	Cp_V2_contig_43085	12.3	3.6	2
DNA-damage-repair/toleration protein DRT100	Cp_V2_contig_25120	12.0	3.6	2
probable inactive purple acid phosphatase 2	Cp_V2_contig_44352	11.5	3.5	2
leucine-rich repeat extensin-like protein 4	Cp_V2_contig_8997	10.5	3.4	2
epidermis-specific secreted glycoprotein EP1-like	Cp_V2_contig_2034	10.5	3.4	2
beta-galactosidase 1	Cp_V2_contig_10156	10.1	3.3	2
putative lipid-transfer protein DIR1	Cp_V2_contig_17417	9.8	3.3	2
low-temperature-induced cysteine proteinase-like	Cp_V2_contig_6578	9.6	3.3	2
subtilisin-like protease SBT1.9	Cp_V2_contig_24584	9.3	3.2	2
cationic peroxidase 1-like	Cp_V2_contig_13540	8.5	3.1	2
F-box/kelch-repeat protein	Cp_V2_contig_38461	8.4	3.1	2

## Data Availability

The mass spectrometry proteomics data have been deposited in the ProteomeXchange Consortium via the PRIDE partner repository with the dataset identifiers PXD026723 (gel-based proteomics) and PXD026733 (gel-free proteomics).

## Supplementary Materials

The following are available online at https://www.mdpi.com/article/10.3390/cells10092295/s1, Figure S1: Phenotype of *C. plantagineum* cell suspension cultures and calli grown in liquid and on solid media supplemented with DCB, Figure S2: Phenotype of *C. plantagineum* cell suspension cultures and calli grown in liquid and on solid media supplemented with IXB, Figure S3: Pie charts showing the distribution, according to their localization, of proteins extracted in the three fractions and analyzed with the gel-free approach, Figure S4: Pie charts showing the distribution, according to their localization, of proteins extracted in the three fractions and analyzed with 2D-DIGE, Table S1: Details of the primers used in this study, with information on the amplicons’ size, R2 and amplification efficiencies (%), Supplementary File S1: Details of the proteins identified as changing significantly with gel-free proteomics, their localization, clustering and abundance in C, DCB-, IXB-habituated cells, Supplementary File S2: Details of the proteins identified with gel-based proteomics, their localization and abundance in C, DCB-, IXB-habituated cells.
