# Supplementary figures and images for: The Cell Wall Proteome of Craterostigma plantagineum Cell Cultures Habituated to Dichlobenil and Isoxaben
Source: Cells. 2021 Sep 2;10(9):2295. doi: 10.3390/cells10092295 (PMC8468770; doi:10.3390/cells10092295)

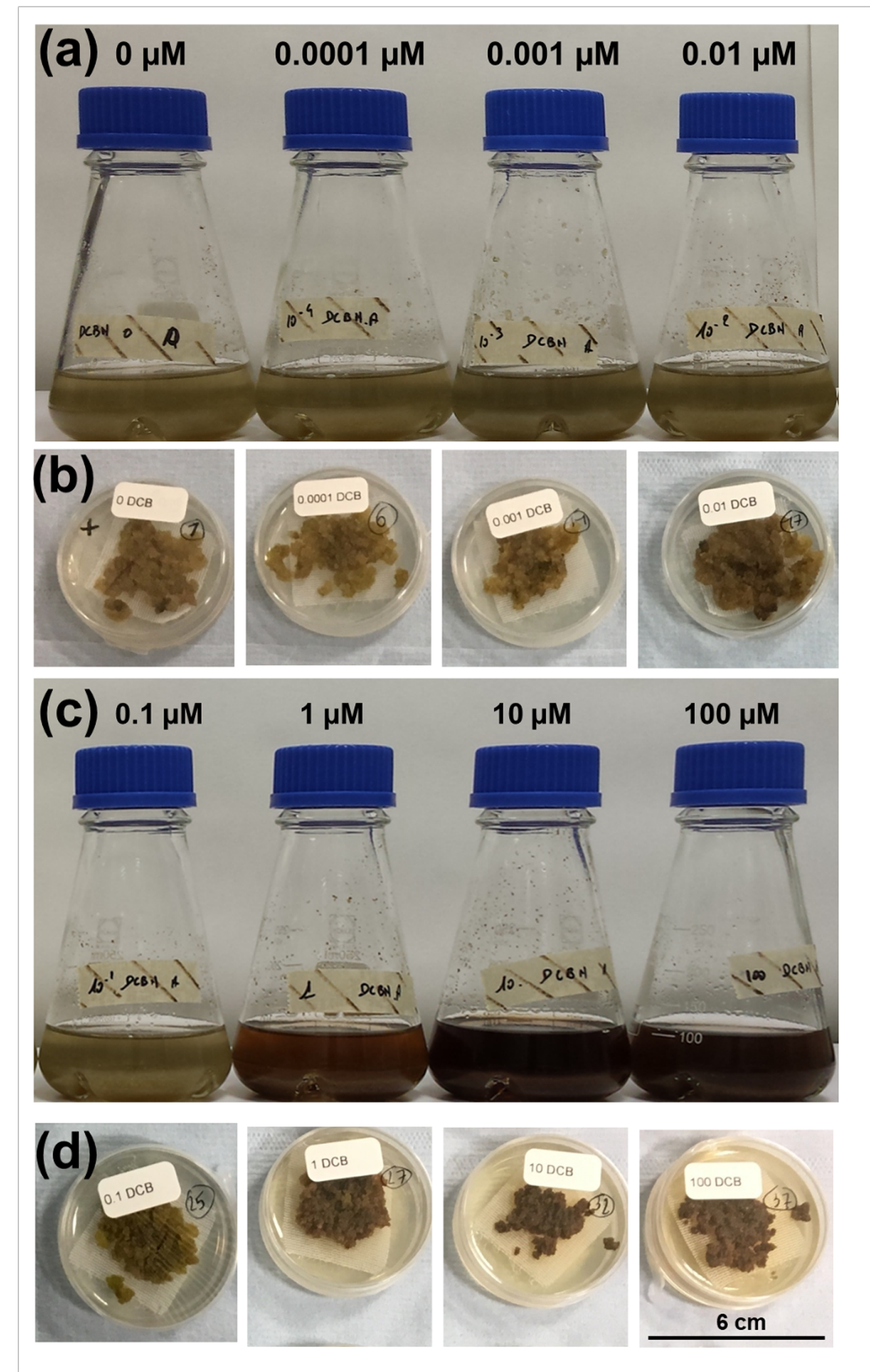

Supplement: Supplementary file 1 [file cells-10-02295-s001.zip › Figure S1 new.tif]

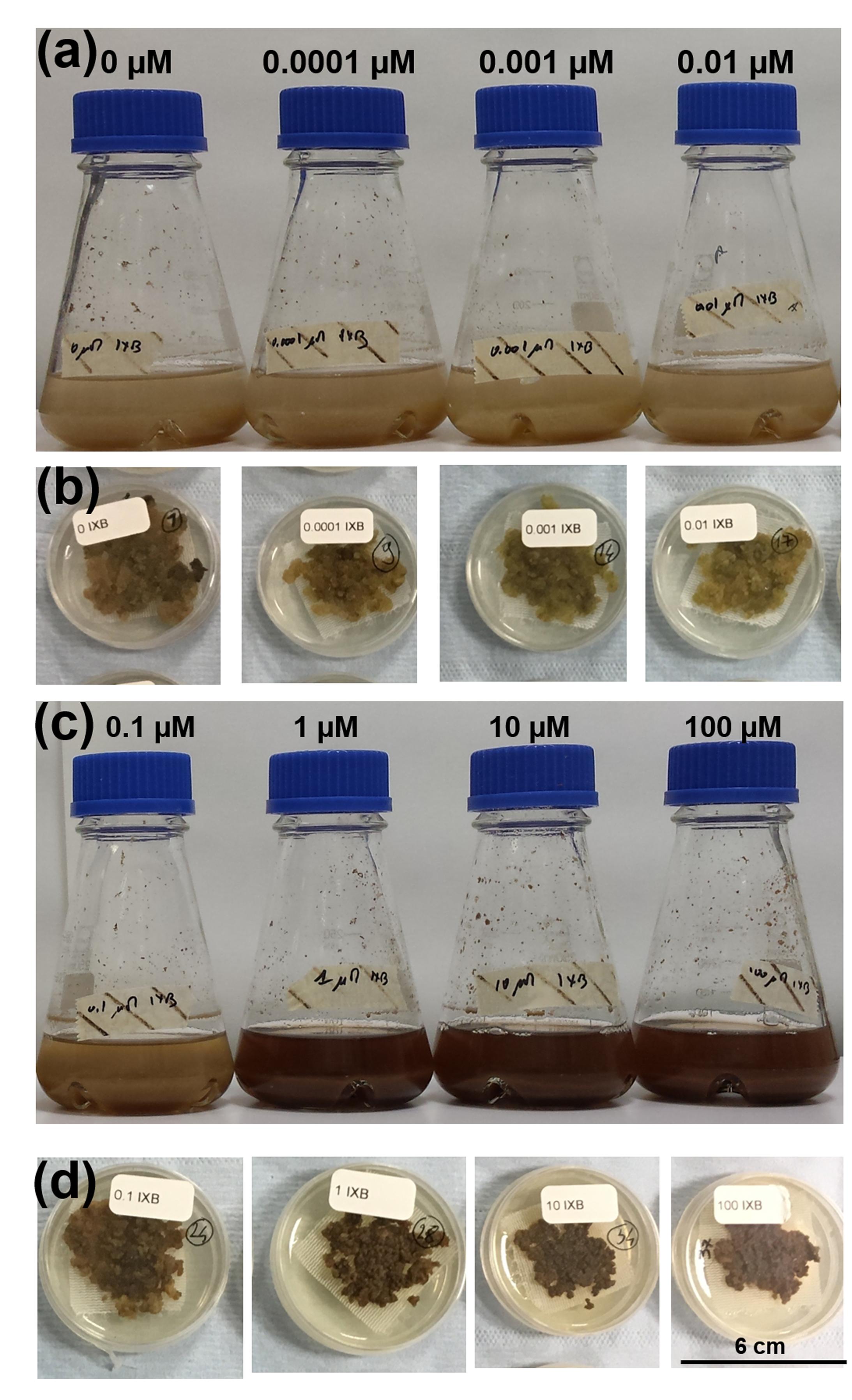

Supplement: Supplementary file 1 [file cells-10-02295-s001.zip › Figure S2 new.tif]

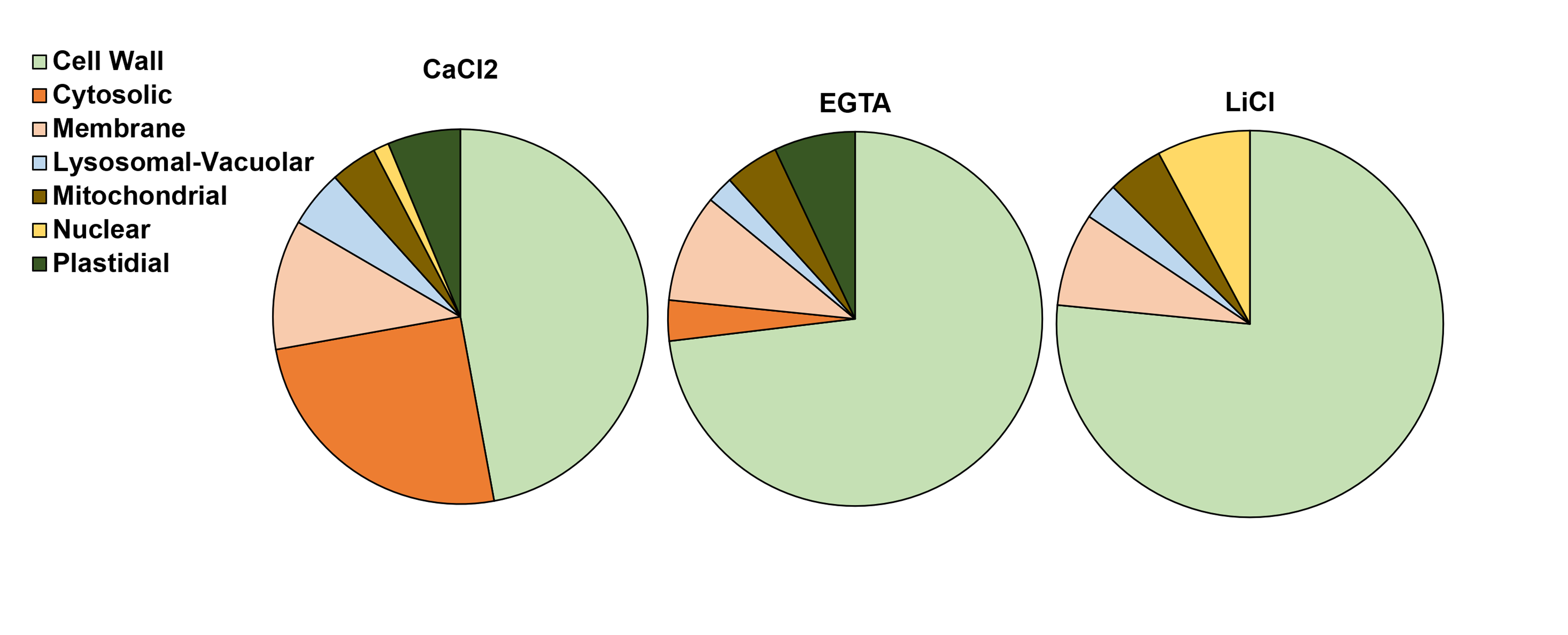

Supplement: Supplementary file 1 [file cells-10-02295-s001.zip › Figure S4 new.tif]
